# Commentary: 1-year risks of cancers associated with COVID-19 vaccination: a large population-based cohort study in South Korea

**DOI:** 10.3389/fpubh.2026.1772406

**Published:** 2026-03-31

**Authors:** Médéa Locquet, Charlotte Beaudart, Jonathan Douxfils, Dominique Roberfroid, Pierre Van Damme, Jean-Michel Dogné

**Affiliations:** 1 Department of Biomedical Sciences, Clinical Pharmacology and Toxicology Research Unit, Namur Research Institute for Life Sciences, Faculty of Medicine, University of Namur, Namur, Belgium; 2 Department of Pharmacy, Clinical Pharmacology and Toxicology Research Unit, Namur Research Institute for Life Sciences, Faculty of Medicine, University of Namur, Namur, Belgium; 3 Department of Medicine, Namur Research Institute for Life Sciences, Faculty of Medicine, University of Namur, Namur, Belgium; 4 KCE, Belgian Health Care Knowledge Centre, Brussels, Belgium; 5 Centre for the Evaluation of Vaccination, University of Antwerp, Antwerp, Belgium

**Keywords:** cancer risk, causal inference, COVID-19 vaccine, critical appraisal, methodological bias

## 1 Introduction

We read with interest the Correspondence by Kim et al. (1) reporting associations between COVID-19 vaccination and 1-year cancer risk in *BMC Biomarker Research*. By contributing to a critical appraisal, our aim is to maintain public and professionals' confidence, in view of current scientific evidence, that COVID-19 vaccination remains a preventive measure for worldwide public health. In the Correspondence, while the large sample and medico-administrative database are strengths, several major methodological issues substantially limit the causal and associational interpretation of findings.

## 2 Temporal biases and inappropriate index date

The vaccinated and unvaccinated groups were assigned different index dates (vaccination date vs. 1 January 2022). This asymmetry introduces calendar time differences in background cancer risk, independent of vaccination. Without time-dependent or delayed-entry survival modeling (2), these temporal biases may have inflated post-vaccination cancer incidence estimates.

## 3 Ambiguity of the treatment effect and analytic design

The 1:4 propensity score matching applied by the authors seems to implicitly target the *Average Treatment Effect on the Treated* (ATT) estimand (i.e., estimating the effect among those who actually vaccinated) rather than the population-level *Average Treatment Effect* (ATE) estimand (i.e., estimating what the outcome would be if the entire population was exposed *vs*. unexposed) (3). Indeed, to better align with worldwide public health policy-level decisions to fight the COVID-19 pandemic, we must consider both the treated and untreated populations, rather than effects limited to those who self-selected for vaccination (as captured by the ATT). The reported hazard ratios thus uniquely reflect associations specific to vaccinated individuals, limiting generalizability to general populations and further raising concerns about differential surveillance bias. Weighted or stratified models would yield more robust, population-relevant estimates.

## 4 Residual confounding and incomplete adjustment

Despite propensity score matching, a slight imbalance persisted in income [standardized mean difference (SMD) = 0.09], a proxy for socioeconomic status that may affect both vaccination and use of cancer screening. Moreover, 77.22% had a prior SARS-CoV-2 infection in both groups, a potential oncogenic exposure (4). Sensitivity analyses excluding infected individuals were not conducted but would have allowed assessment of findings consistency without potential confounding carcinogenic effects of SARS-CoV-2 infection. Additionally, substantial unmeasured residual confounding remains (e.g., smoking, alcohol, comorbidities, family history, genetic susceptibility, and screening frequency).

## 5 Surveillance bias

The observed “increased” cancer risk may reflect opportunistic detection of pre-existing subclinical malignancies among the vaccinated individuals, who are likely to have higher healthcare use and surveillance. South Korea's extensive national screening programs for thyroid, breast, gastric, and colorectal cancers could amplify this effect (5–8).

## 6 Multiple testing and false positives

A total of 30 site-specific and subgroup analyses were performed without correction for multiple comparisons, increasing the risk of type I error and subsequent probable false associations due to chance alone.

## 7 Biological implausibility

One-year solid tumor carcinogenesis does not seem biologically plausible. The reported associations are thus more consistent with study design biases and residual confounding than with causal vaccine effects. The apparent “protective” association for leukemia in the booster subgroup (i.e., probable type I error) further supports a very cautious interpretation. A lag period excluding cancers diagnosed within 6–12 months post-vaccination, at least, would help reduce such bias and better control for cancer latency. Furthermore, longer-term studies (at least 10 years) are needed to explore the authors' hypothesis.

## 8 Implications and recommendations

According to the Grading of Recommendations Assessment, Development and Evaluation (GRADE) criteria (9), this study provides low-quality evidence, as the observational design, high risk of bias, and important residual confounding substantially reduce confidence in the estimated association between COVID-19 vaccination and cancer risk.

We therefore recommend a re-analysis emulating a target trial framework (10), incorporating time-varying exposure, appropriate comparison groups, longer follow-up, exclusion of early diagnoses, healthcare utilization matching, statistical multiple testing corrections, and rigorous sensitivity analyses.

In conclusion, the findings of Kim et al. should be interpreted with caution, as causation cannot be proven using such a study design. Furthermore, the previously highlighted potential source of multiple biases also calls for reconsideration of the reported associations. We thank the authors for hypothesis generation and transparency while encouraging further analyses following robust epidemiological standards before communicating such sensitive scientific findings. Indeed, high responsibility is needed to clearly distinguish between association and causation and to interpret findings within their appropriate biological and methodological context. Overinterpretation or misrepresentation of such a study could unjustifiably contribute to vaccine hesitancy that could lead to preventable, sometimes life-threatening, adverse health outcomes (11). Scientific responsibility is required to maintain such caution and nuance, crucial to avoid misinterpretation or inappropriate use of scientific evidence, in the current context of declining confidence and hesitancy in vaccination, however largely recognized as a major preventive measure to maintain worldwide public health (12). Subsequently, the findings reported by Kim et al. should not be used to inform or guide clinical practice or public health policy in the absence of robust associational or causal evidence, given the substantial methodological limitations identified.
